# Pluronic F-127/Silk Fibroin for Enhanced Mechanical Property and Sustained Release Drug for Tissue Engineering Biomaterial

**DOI:** 10.3390/ma14051287

**Published:** 2021-03-08

**Authors:** Jina Youn, Joo Hee Choi, Sumi Lee, Seong Won Lee, Byung Kwan Moon, Jeong Eun Song, Gilson Khang

**Affiliations:** 1 Department of Bionanotechnology and Bio-Convergence Engineering, Jeonbuk National University, 567 Baekje-daero, Deokjin-gu, Jeonju-si, Jeonbuk 54896, Korea; yjina@jbnu.ac.kr (J.Y.); zooheechoi@jbnu.ac.kr (J.H.C.); sumilee@jbnu.ac.kr (S.L.); tjddnjs5140@jbnu.ac.kr (S.W.L.); songje@jbnu.ac.kr (J.E.S.); 2 Department of PolymerNano Science & Technology and Polymer Materials Fusion Research Center, Jeonbuk National University, 567 Baekje-daero, Deokjin-gu, Jeonju-si, Jeonbuk 54896, Korea; byungkwan.moon@celltrionchem.com

**Keywords:** hydrogel, tissue engineering, pluronic F-127, silk fibroin

## Abstract

Herein, an injectable thermosensitive hydrogel was developed for a drug and cellular delivery system. The composite was prepared by facile physical mixing of pluronic F-127 (PF) and silk fibroin (SF) in an aqueous solution. The chemical structure, transparency, viscosity, injectability, degradation kinetic, cumulative release of dexamethasone (Dex), a type of corticosteroid drug, and size distribution of the fabricated hydrogels were characterized. Cytotoxicity of the hydrogels was also studied to verify the biocompatibility of the hydrogels. The addition of a proper amount of SF to PF not only improved the mechanical strength but also decreased the degradation rate which improved the fast rate release of hydrophobic drugs. The cytotoxicity of the hydrogel decreased when SF was added to PF in a proper amount. Overall, the results confirm that the composite of PF and SF can be a promising cell and drug delivery system for future application in tissue engineering and regenerative medicine.

## 1. Introduction

One of the aims of tissue engineering (TE) is to implant alternative substances such as cells, drugs, biomolecules, and growth factors into the damaged or injured tissue for therapeutic purposes [1,2,3,4,5,6]. Hydrogel-based TE treatment is one of the most promising regeneration strategies and has received considerable attention from various researchers in biomedical fields. Hydrogel mimics salient components of native extracellular matrices (ECM) and has similar mechanical properties to those of many soft tissues [7,8,9]. The application of hydrogels has proven to be effective in a range of cell culture applications to a delivery system [10,11,12]. Advanced hydrogel-based biomaterials are continuously proposed and studied.

Among various types of hydrogels, thermosensitive hydrogels possess great potential for a wide range of TE applications [12,13,14,15]. The sol–gel transition of thermosensitive hydrogels can appear at a physiological temperature (~37 °C). Pluronic F-127 (PF) is one of the promising thermosensitive hydrogels which is used for various application [16,17,18]. PF is composed of an A-B-A non-ionic tri-block copolymer structure where A is a hydrophilic poly (ethylene oxide) (PEO) and B is a hydrophobic poly (propylene oxide) (PPO) [19]. The merits of PF are that it is biocompatible, non-toxic, and can readily form a hydrogel at a specific temperature [20]. Further, PF forms a micelle during the gelation process which allows encapsulation of hydrophobic drugs in the core of the micelle and forms a hydrophilic interaction with various biological factors [21,22,23]. However, in the physiological conditions, the hydrogel structure is unstable due to the low molecular weight and poor mechanical properties. These drawbacks cause rapid decomposition and cause the burst release of the drug [24]. To compensate the poor mechanical and physicochemical properties of PF, high concentrations of PF can be used but may result in undesirable side effects in vivo [25]. To overcome these problems, different types of polysaccharide and collagen-derived polymers such as hyaluronic acid, alginate, chitosan, gellan gum, and gelatin have been applied with PF to enhance the stability and physicochemical properties of PF through the intramolecular interaction [16,26,27,28,29,30]. A facile blending with various types of matrix exhibited a promising application as an injectable hydrogel for drug and cell delivery. Studies on a PF micelle which was physically blended with silk-related protein were also performed in previous papers, but no studies have been conducted on the possibility of an injectable device [31,32,33].

**Scheme 1:**
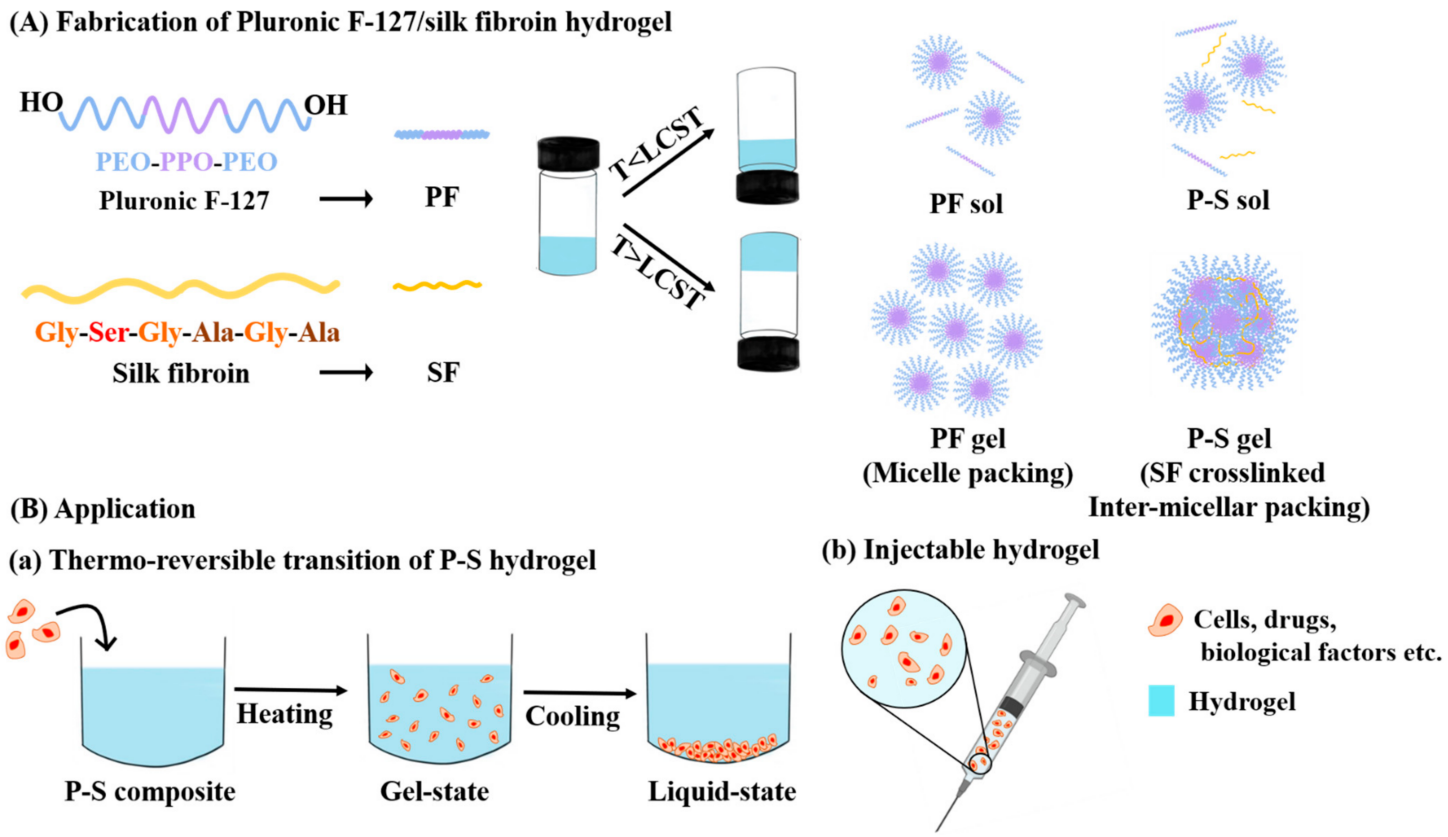
(**A**) Fabrication method and mechanism of Pluronic F-127 (PF)/silk fibroin (SF) (P–S) hydrogel; (**B**) Application of P–S hydrogel: (**a**) A thermo-reversible transition for 3-dimensional (3D) culture and (**b**) Injectable device for cells, drugs, and biological factors etc. delivery.

Herein, silk fibroin (SF) extracted from the Bombyx mori cocoon is used to compensate for the mechanical properties and stability of PF. SF is widely reported to be biocompatible and biodegradable, and to have high cell adhesion moieties, low antigenicity, superior mechanical properties, and ease of handling [34,35,36,37]. For these advantages, SF is actively used to form a three-dimensional (3D) scaffold, or for a drug delivery system [38]. However, SF requires rather harsh external stimuli such as pH, ultrasound, chemical modification, and chemical crosslinking to obtain an SF-based hydrogel [39,40]. To solve the limitations of both PF and SF, non-toxic physical crosslinking was applied in this study. It was hypothesized that the mixture PF and SF may enhance the mechanical properties of PF by inter-micellar packing through the physical crosslinking of SF and form a thermo-reversible injectable hydrogel for tissue engineering biomaterial (Scheme 1). The various amounts of SF were loaded in PF, and the characterization was carried out with FT-IR, a transparency test, gross observation, a size distribution test, a viscosity study, injectability, degradation, and release studies, and a cytotoxicity test.

## 2. Materials and Methods

### 2.1. Preparation SF Solution

An amount of 10 g of the silkworm cocoon was cut and boiled in a solution containing 0.02 M sodium carbonate (Na_2_CO_3,_ Showa Chemical Industry Co., Tokyo, Japan) to remove the sericin. After boiling for 30 min, it was washed with distilled water and put in a 60 °C oven to remove water. An amount of 7 g of the dried silk was mixed with 9.3 M lithium bromide (LiBr, Kanto Chemical, Tokyo, Japan) and stored at 60 °C for 4 h. When the silk completely dissolved, it was dialyzed in distilled water for 72 h using a Snakeskin^TM^ Dialysis Tubing (3500 MWCO, Thermo Fisher Scientific, Waltham, MA, USA) to remove LiBr.

### 2.2. Fabrication of the Hydrogels

PF powder (Sigma-Aldrich, St. Louis, MO, USA) with an amount of 20% (w/v) was stirred at 4 °C in distilled water. SF solution was blended with PF solution with the final volumes of 0%, 0.5%, 1%, and 2% (w/v), which are specified as PF, P–S0.5, P–S1, and P–S2, respectively, and was stirred at 4 °C for 3 h. The fabricated hydrogel solutions were stored at 4 ℃ until further characterization.

### 2.3. Characterization

#### 2.3.1. Chemical Structure Analysis

The chemical structures of SF, PF, and Pluronic F-127/silk fibroin (P–S) hydrogels were measured by attenuated total reflectance-Fourier transform infrared (ATR-FTIR, Perkin Elmer, Boston, MA, USA) at the wavelength range of 400–4000 cm^−1^. All the samples were lyophilized at −84 °C and a vacuum gauge of 5 m Torr using a freeze dryer (ilShinBioBase Co., Ltd., Dongducheon, Korea) to fully remove moisture.

#### 2.3.2. Weight Loss Ratio

The hydrogels with an amount of 500 μL were placed in 1.5 mL microtubes and gelation was induced by placing them in a 37 ℃ condition. The weight of the solidified hydrogel was measured (*W_i_*). Then, 500 μL of phosphate-buffered saline (PBS, Gibco, Thermo Fisher Scientific, Waltham, MA, USA) was added and the samples were stored at 37 °C. At the specific time points, the hydrogels were centrifuged at 2000 rpm for 15 min at 37 °C using a Micro High Speed Centrifuge (Micro 17TR, Hanil Scientific Inc., Gyeonggi, Korea). The supernatant was removed and the remaining weight was weighed (*W_t_*). The weight loss of the gels was calculated the following Equation (1) [41].

(1) $$\mathrm{Weight}\;\mathrm{loss}\;(\%)\;=\;\frac{W_{i}-W_{t}}{Wi}\times 100\;\left(\%\right)$$

#### 2.3.3. Optical Intensity

The transparency of the P–S hydrogels was measured at 4 and 37 °C by gross observation and spectrum analysis, using a Synergy MX spectrophotometer (BioTek, Vernusky, VT, USA) at a wavelength range of 380–780 nm. The transmittance of the specimens was calculated following Equation (2).

(2) $$\mathrm{Absorbance}\;\left(A\right)=2-\log\%\mathrm{Transmittance}\;\left(T\right)$$

#### 2.3.4. Viscosity Evaluation

The viscosity and gelation temperature of P–S was measured by a viscometer (AMETEK Brookfield, Middleboro, MA, USA). The initial temperature was set at 18 °C and each P–S solution was measured by adding 8 mL to the viscometer. The temperature was gradually increased and viscosity was measured at various temperatures. The router speed was set at 1 rpm, and cone and plate spindles (LV-04 spindle, AMETEK Brookfield, Middleboro, MA, USA) were applied for this study.

#### 2.3.5. Injection Force Test

The injection force of the fabricated hydrogel was studied by following the previous reported study with a slight modification [42]. The prepared samples were aspirated in a 1 mL syringe (Kovax-Syringe, Korea Vaccine Co., Ltd., Seoul, Korea) with an amount of 500 μL. The syringes were capped with a $6G\frac{1}{2}\prime$ needle and stored at RT for 5 min to solidify the hydrogel solution. The samples were placed on a custom-designed bracket and the injection force test was carried out with a Texture Analyzer (Version 1, 2013, Food Technology Corporation, West Sussex, VA, USA) at a speed of 20 mm/min and load cell of 20 N. The needle was submerged in the PBS solution to create a similar condition to in vivo injection. The experiment was performed at 37 °C and 4 parallel measurements was carried out.

#### 2.3.6. Release Study

The release property of PF and P–S was conducted using dexamethasone (Dex, Sigma-Aldrich, St. Louis, MO, USA), a type of corticosteroid drug. First, 20% (w/v) PF solution was fabricated, and SF solution was blended with PF solution with the final volumes of 0%, 0.5%, 1%, and 2% (w/v) and stirred at 4 °C. Then, Dex was dissolved in an amount of 1 mg/mL. The manufactured hydrogel solution was transferred into a Snakeskin^TM^ Dialysis Tubing (Thermo Fisher Scientific, Waltham, MA, USA) in an amount of 3 mL. The samples were incubated in 15 mL of PBS at 37 °C. At specific time points (0.5, 1, 3, 5, and 8 h), all of the extracted solutions were transferred to a new conical tube and fresh PBS was added to the samples. The released Dex was analyzed by a microplate reader (Synergy MX, BioTek, Vernusky, VT, USA) at an absorbance of 241 nm.

#### 2.3.7. Size Analysis of P–S Hydrogel

The micelle size and packing of the hydrogel were characterized at 37 °C using a particle size analyzer (90Plus, Brookhaven Instrument Corp., Holtswille, NY, USA) at a scattering angle of 90°. The concentration of PF and P–S hydrogels was 1.875% (w/v).

### 2.4. In Vitro Study

#### Cytotoxicity Evaluation

The cytotoxicity of the P–S hydrogels was characterized by the extraction test reported in the previous study with a slight modification [27]. An amount of 9 mL of RPMI (Gibco, Thermo Fisher Scientific, Waltham, MA, USA) supplemented with 10% fetal bovine serum (FBS, Gibco, Thermo Fisher Scientific, Waltham, MA, USA) and 1% penicillin/streptomycin (PS, Gibco, Thermo Fisher Scientific, Waltham, MA, USA) was carefully added in 1 mL of solidified hydrogel samples. The prepared samples were incubated in a 37 °C water bath for 24 h. Autoclaved latex was chopped into pieces for the positive control (2.5 cm^2^/mL) and was incubated with 10 mL of RPMI medium for 24 h in a 37 °C water bath. Then, the extracted supernatant was filtered through a 0.45 µm pore size filter (Millex^®^ Syringe Filters, Merk Millipore, Darmstadt, Germany). NIH/3T3 mouse embryo fibroblast (National Institute of Health, KCLB2165, Korean Cell Line Bank, Seoul, Korea) was used for the cytotoxicity evaluation. The cells were cultured on 96-well plates (n = 6, 2 × 10^3^ cells/well) in RPMI cell culture medium. The cells were incubated for 24 h at 37 °C in a 5% CO_2_ incubator. The cell culture medium was removed after 24 h and the samples were added and stored under standard culture conditions (5% CO_2_ and 37 °C). At a specific time point, MTT (3-[4,5-dimethylthiazol-2-yl]-2,5-diphenyltetrazolium bromide; thiazolyl blue, 5 mg/mL in PBS, Amresco, Dallas, TX, USA) solution was treated and the plates were incubated for 1 h in a cell culture incubator. The supernatant was removed and dimethyl sulfoxide (DMSO, Samchun chemical, Seoul, Korea) was added to dissolve the formazan crystal. The absorbance of 570 nm was measured with a microplate reader (Synergy MX, Biotek, Vernusky, VT, USA). All the groups were normalized with the negative control (RPMI medium). All the groups were normalized with the negative control (cell culture media).

## 3. Results and Discussion

### 3.1. Fabrication and Chemical Structure Evaluation

**Figure 1:**
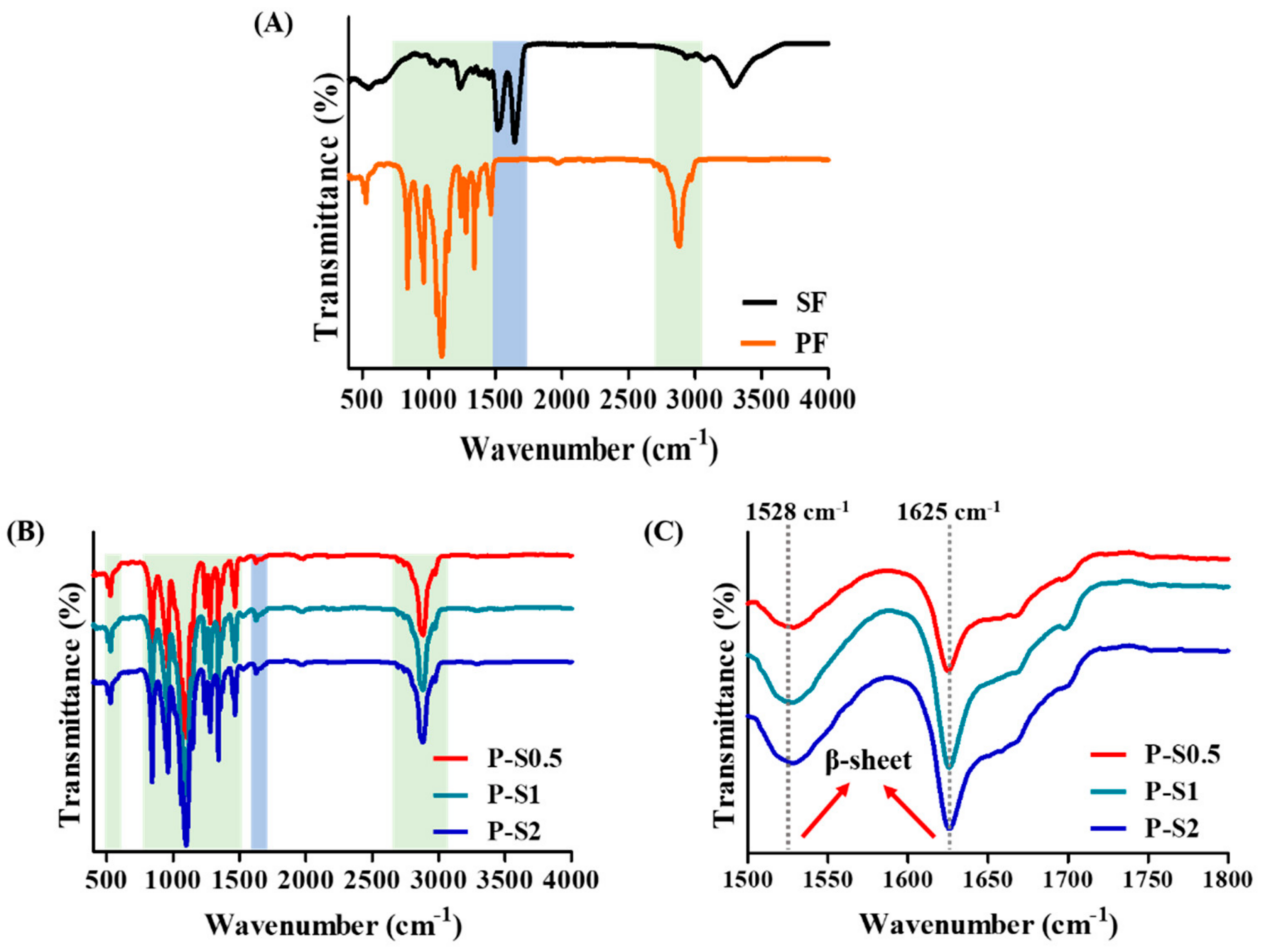
ATR-FTIR evaluation of (**A**) lyophilized SF solution and PF hydrogel and (**B**) Lyophilized P–S hydrogels measured at the wavelength range of 400–4000 cm^−1^ and (**C**) Magnified transmittance of P–S hydrogels at 1500–1800 cm^−1^ wavelength.

The 20% (w/v) PF concentration in P–S hydrogels is an adequate and widely used formulation for use as an injectable hydrogel [26]. At physiological temperature, the PF copolymer less than 15% (w/v) becomes a sol state and appears as a low-viscosity liquid; however, the 20% PF copolymer exists in a gel state [43]. When the concentration of PF is too high, side effects exist when used in the body [44]. The chemical composition of PF, SF, and the interaction of the P–S composite was identified by using ATR-FTIR (Figure 1). The specific peaks of PF were exhibited at 2882 (C-H stretching vibration), 1342 (in plane O-H band), and 1098 cm^−1^ (C-O-C stretching) (Figure 1A) [45]. In SF, the characteristic peaks were observed at 3334 (N-H stretching from the peptide bond of protein), 1646 (amide Ⅰ), 1517 (amide Ⅱ), and 1236 cm^−1^ (amide Ⅲ) (Figure 1A) [46]. The P–S composite showed specific peaks of PF (represented in the green box) which did not display any shifting. The specific peaks of amide Ⅰ and amide Ⅱ of the P–S hydrogels appeared at 1625 and 1528 cm^−1^ (represented in the blue box), respectively (Figure 1B,C). The shifting of the wavenumber may be due to the intermolecular interaction among PF and SF and also the formation of β-sheets from the water absorbance of PF within SF [32]. New peaks did not appear which proves that the hydrogel was manufactured based on physical mixing of PF and SF.

### 3.2. Viscosity and Injection Force Analysis

**Figure 2:**
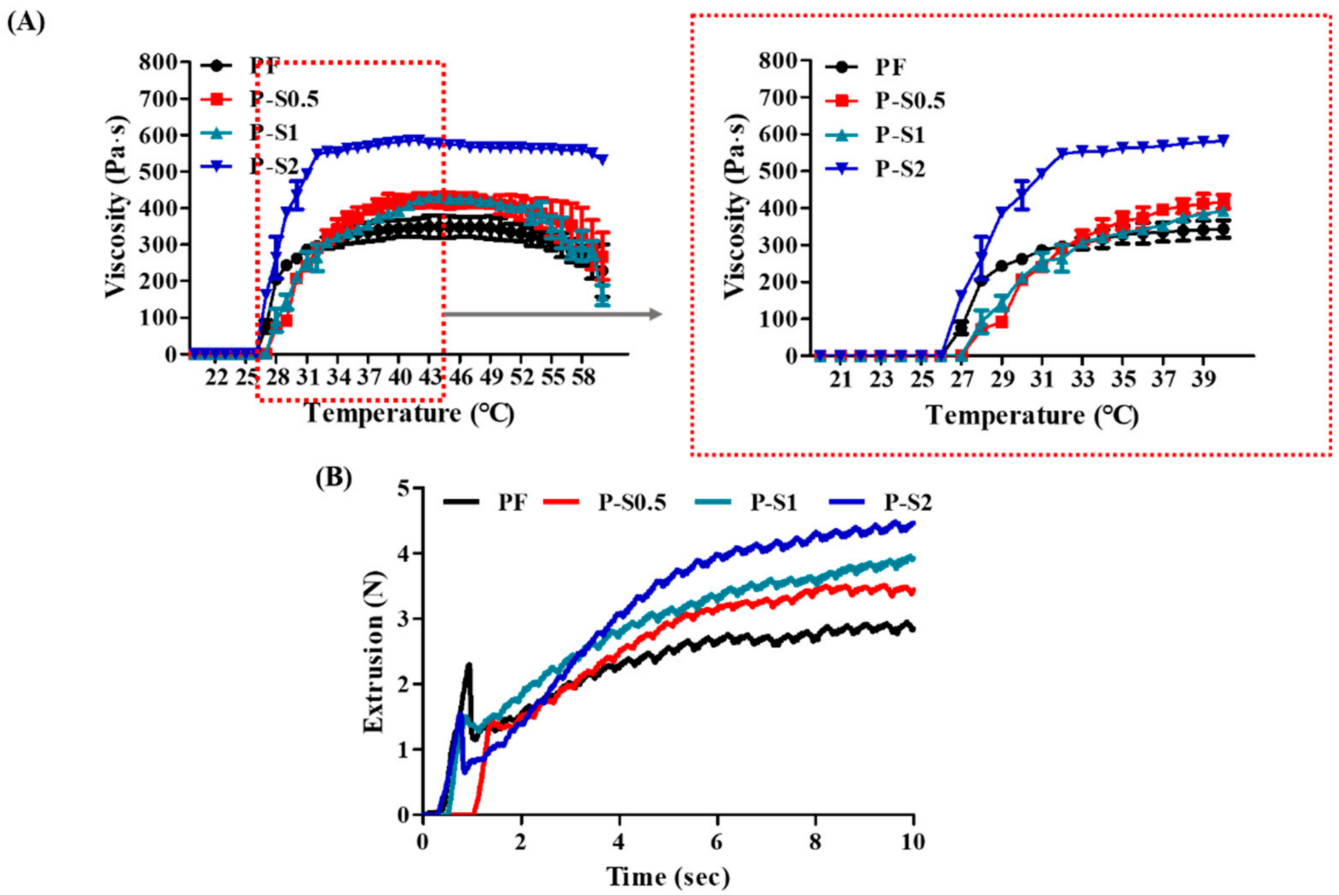
(**A**) Viscosity analysis by gradually increasing the temperature from 18 to 60 °C. (**B**) Extrusion test of the hydrogels analyzed at room temperature at a speed of 20 mm/min and load cell of 20 N.

The gelation temperature of the hydrogels and mechanical properties of the hydrogels were measured by a viscosity study and an injection force test. The gelation temperature of the hydrogels showed at ~27.3, ~27.3, ~27.4, and ~27.4 °C in PF, P–S0.5, P–S1, and P–S2, respectively (Figure 2A). The final viscosity of the P–S hydrogels at a temperature above 37 °C showed a higher storage modulus when compared to PF. It can be seen that the temperature-induced phase transition behavior of PF hydrogels was not significantly affected by the addition of SF. However, the maximum viscosity value of each sample was increased by adding SF. It is suspected that the intermolecular interaction among PF and SF induced higher mechanical properties when the P–S composite reached a specific temperature. The injection property is an important property of the hydrogel to be applied in tissue engineering and regenerative medicine (Figure 2B). The PF group showed the lowest injection force, while the SF-loaded hydrogels showed a higher injection force. As a higher amount of SF was contained, the mechanical properties increased which required a higher force to inject, but it was sufficient to inject.

### 3.3. Gross Image and Transparency

**Figure 3:**
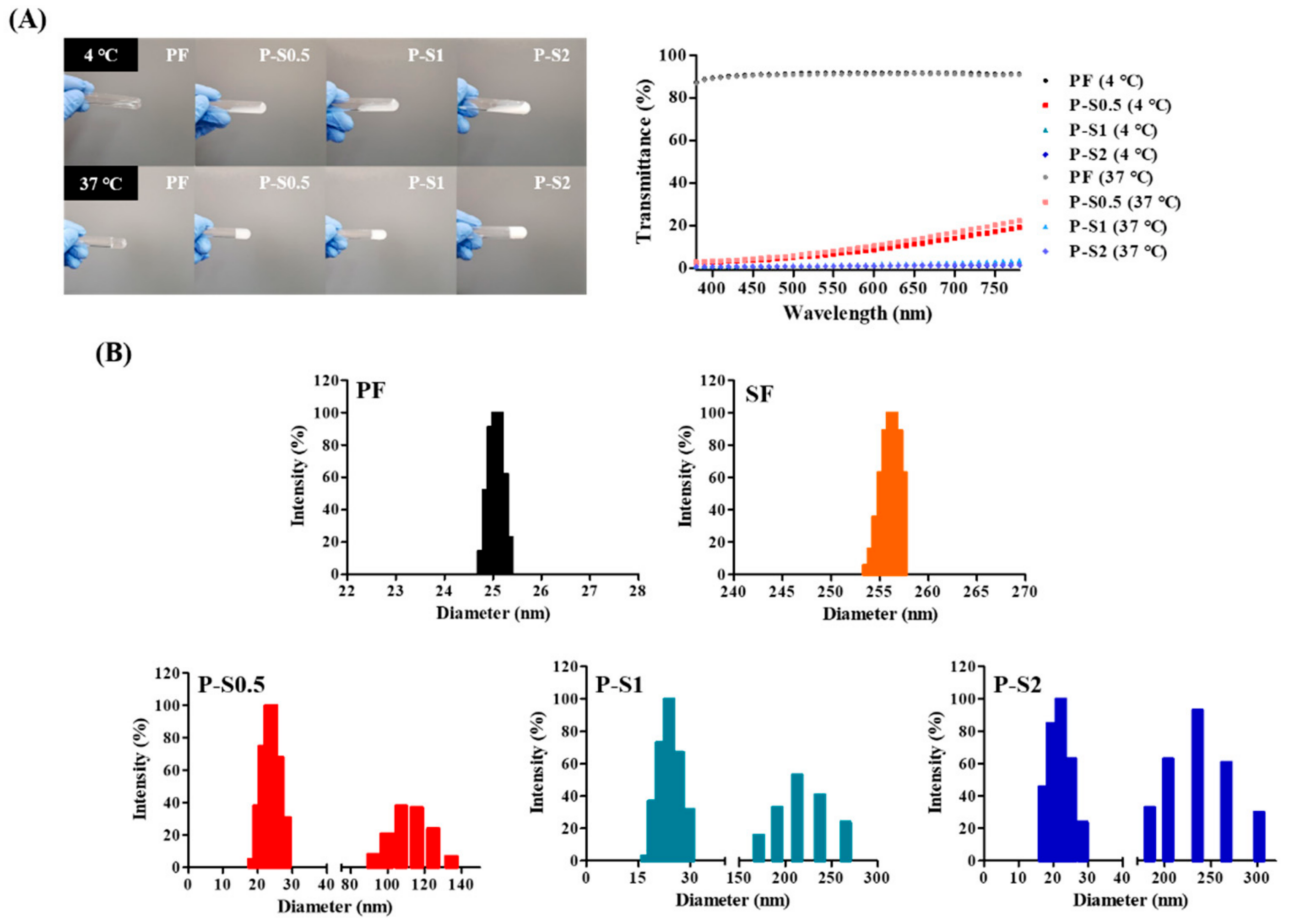
(**A**) Gross images and transparency results in the visible range wavelength of 380–780 nm. (**B**) Size distribution of PF, SF, P–S0.5, P–S1, and P–S2 at 37 °C.

The gross image and transparency of the fabricated hydrogels were characterized at 4 and 37 °C (Figure 3A). All the groups exhibited a liquid-like property at 4 °C and a solid-like property at 37 °C, which confirms the thermo-responsiveness of the hydrogels. The PF groups showed excellent transparency both at 4 and 37 °C. The P–S groups showed lower transparency when compared to the PF group, which may be due to the intermolecular interaction in the matrix. Moreover, the lower transparency of the P–S groups may be due to the formation of β-sheets in SF by the absorbance of the water from the hydrophilic part in PF, inducing a conformational change of SF from a random coil to a β-sheet structure [32]. The higher content of SF in the PF solution exhibited lower transparency from the existence of a higher content of β-sheets. At 37 °C, although the difference was insignificant, the transparency of the P–S group showed lower transparency than that in the liquid state. It was believed that this phenomenon occurred due to the micellar packing of PF from the physical crosslinking of SF. To confirm the intermolecular interaction among PF and SF, the particle size of the PF and P–S groups was analyzed at 37 °C (Figure 3B).

The PF group showed ~26 nm in particle size at 37 °C, while the SF group exhibited a particle size of ~265 nm. P–S groups exhibited the particle size at two divided areas. The P–S0.5 group showed a particle size of ~23 and ~115 nm, the P–S1 group displayed a particle size of ~24 and ~230 nm, and the P–S2 group exhibited a particle size of ~22 and ~235 nm. The first section of the particle size of the P–S groups may be due to the micelle formation of PF. The particle size of the second section of the P–S groups may be attributed to the inter-micellar packing through the intermolecular interactions among SF and PF. The size of the particle increased as the SF content was higher. In this regard, the transparency of the P–S hydrogel groups may have decreased at 37 °C when compared to the transparency of 4 °C due to the densely packed micelles. However, as the content of SF increased, the particle size exhibited a broader range. It is suspected that above the critical point, SF is dispersed in the matrix and forms an intermolecularly interacting SF matrix rather than working as a micelle physical crosslinker.

### 3.4. Weight Loss and Release Study

**Figure 4:**
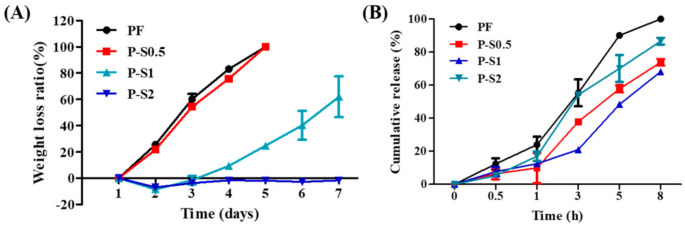
(**A**) Weight loss ratio of the hydrogels analyzed for 7 days. (**B**) Cumulative release kinetics analyzed for 48 h (values are means ± SD, n = 4).

The weight loss ratio of hydrogels was analyzed for 7 days (Figure 4A). The PF group and P–S0.5 group showed a fast rate of degradation and were completely dissolved on day 5 of the experiment. On the other hand, P–S1 and P–S2 hydrogels maintained the structure for 3 days of the experiment. The P–S1 group started to degrade on day 4 of the experiment and on day 7, the samples remained at 62.01 ± 15.59% of the initial weight. The P–S2 group did not show a degradation kinetic for 7 days. This confirms that incorporation of SF enhances the physicochemical property of PF by physical crosslinking. However, it is suspected that the enhanced physicochemical property of P–S2 occurred due to the increased interaction among SF, rather than the inter-micellar physical crosslinking. The cumulative release of the drug was also confirmed (Figure 4B).

PF can be applied as a drug delivery vehicle but exhibits burst release at the initial time point [26,47]. However, it was predicted that rapid release can be prevented by inter-micellar packing. The PF group showed the fastest release kinetic when compared to the P–S groups, and the entire Dex was released within 8 h. The reason for the fast release of PF is due to the hydrophilicity of the PF micelles [47,48]. The P–S0.5 and P–S1 groups showed slower release kinetics when compared to PF. This may be due to the barrier effect of the inter-micellar packing which delayed the release of the drugs [49]. Further, the mesh size of the hydrogel can be predicted from the mechanical properties. The crosslinking density of the hydrogel increases as the mechanical properties are higher and the mesh size of the hydrogel matrix decreases [50,51]. Through this, it can be predicted that the reduced mesh size slowed down the drug release kinetic of the P–S hydrogel. On the other hand, the P–S2 group exhibited a faster release kinetic when compared to P–S0.5 and P–S1, which may be due to the dispersed SF rather than the formation of micelle stacking which coincides with the size distribution result [52]. The release study shows that the proper amount of SF not only improved the overall mechanical properties but also formed an obstruction that prevents the burst release of PF.

### 3.5. Cell Viability and Cytotoxicity Test

**Figure 5:**
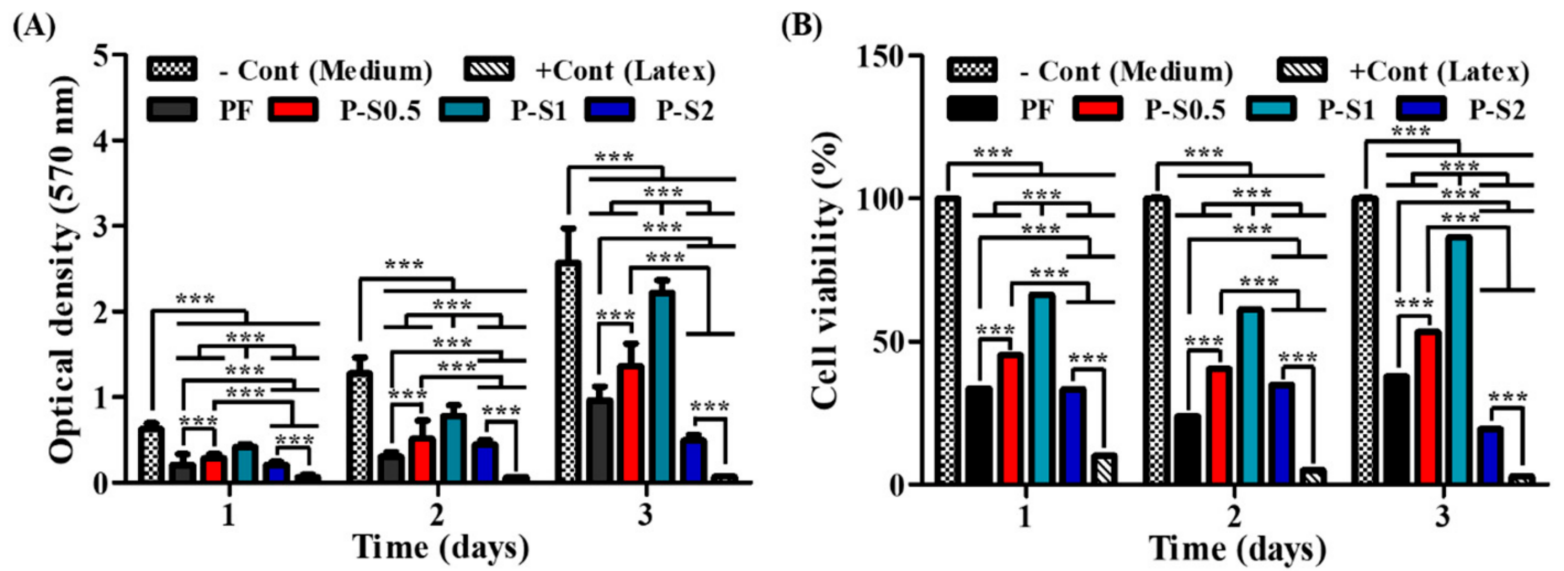
Cytotoxicity test of hydrogels analyzed for 72 h by using the medium extraction method. (**A**) Cell viability expressed by optical density. (**B**) Representation of the percentage of P–S hydrogels assuming the cell viability of the negative control group at 100% (NIH 3T3 cell line was used for this study) (values are means ± SD, n = 8, *p* < 0.001 (***)).

The biocompatibility of the fabricated material was confirmed by an extraction test (Figure 5). In order to fabricate a PF hydrogel with sufficient mechanical properties, the concentration should be above 15%. However, although it may depend on the type of cell, the cytotoxicity increases when the concentration is too high. This is because high concentrations may disrupt the cell membrane [53,54]. The number of NIH 3T3 cells increased in both PF and P–S groups when the time passed (Figure 5A). However, the PF group showed only 37.68 ± 0.16% of cell viability compared to the negative control on day 3 of the experiment (Figure 5B).

It is believed that the high amount of decomposition factors of PF existed in the extracted medium solution from the fast degradation rate of PF which disrupted the membrane of the cells [53,54]. The viability of the cells increased in the P–S0.5 and P–S1 groups. This may be due to the enhancement of the physical crosslinking of the hydrogel matrix which inhibited the PF release. In particular, the P–S1 group exhibited 86.52 ± 0.15% of cell viability which significantly enhanced the biocompatibility of PF. However, the cell viability was 19.27 ± 0.07% in the P–S2 groups, which may be due to the release of a residual SF matrix to the cells [55]. This illustrates the importance of the incorporation of a proper amount of SF to enhance the biocompatibility.

## 4. Conclusions

In this study, a P–S hydrogel was proposed as an effective biomaterial for application in tissue engineering and regenerative medicine. PF provided a platform for the entire support. SF supported enhancing the intermolecular interaction of micelles by physical crosslinking. P–S hydrogels were prepared by a simple physical mixing without any chemical modification. Thermo-reversible P–S hydrogels showed sol–gel transition at 4 and 37 °C, which shows the potential of these gels as injectable hydrogels. The rapid weight loss and cumulative release of pure PF were improved by the intermolecular interaction of SF. Overall, the P–S hydrogels incorporated with the proper amount of SF showed enhanced mechanical and physicochemical properties when compared to PF. Moreover, the biocompatibility of the PF was significantly enhanced in the P–S hydrogel when compared to the pristine PF. The results suggest that the P–S hydrogel incorporated with biological factors or drugs may provide stable release in vivo. Further, cells can be encapsulated in P–S hydrogels, which can be applied in 3D culture systems, or used for cell delivery vehicles. Overall, the P–S hydrogel is a promising injectable hydrogel for future tissue regeneration application.

## Data Availability

Data is contained within the article.
